# Design and Implementation of a Self-Powered Smart Water Meter

**DOI:** 10.3390/s19194177

**Published:** 2019-09-26

**Authors:** Xue Jun Li, Peter Han Joo Chong

**Affiliations:** Department of Electrical and Electronic Engineering, Auckland University of Technology, Auckland 1010, New Zealand; peter.chong@aut.ac.nz

**Keywords:** water meter, smart city, automatic meter reading, energy harvesting, flow measurement

## Abstract

Smart cities require interactive management of water supply networks and water meters play an important role in such a task. As compared to fully mechanical water meters, electromechanical water meters or fully electronic water meters can collect real-time information through automatic meter reading (AMR), which makes them more suitable for smart cities applications. In this paper, we first study the design principles of existing water meters, and then present our design and implementation of a self-powered smart water meter. The proposed water meter is based on a water turbine generator, which serves for two purposes: (i) to sense the water flow through adaptive signal processing performed on the generated voltage; and (ii) to produce electricity to charge batteries for the smart meter to function properly. In particular, we present the design considerations and implementation details. The wireless transceiver is integrated in the proposed water meter so that it can provide real-time water flow information. In addition, a mobile phone application is designed to provide a user with a convenient tool for water usage monitoring.

## 1. Introduction

A smart city was proposed as an urban development vision to integrate state-of-the-art technologies, such as information and communication technology (ICT), and Internet of Things (IoT) in a secure fashion of managing a city [1]. The management includes residential/commercial buildings, schools, libraries, transportation, hospitals, power generation/distribution networks, water supply networks, waste collection/transportation/disposal, law enforcement and other community services. A smart city promotes the usage of real-time information and provides the interactive platform for people to manage the city with significantly improved efficiency as compared to the traditional way. In general, a smart city has the following three features: (i) *Instrumentation Intelligence*—efficient use of physical infrastructure through real-time informatics [2] to support a strong and healthy economic, social and cultural development; (ii) *Collective Intelligence*—interactive engagement with residents in local governance and decision-making processes; and (iii) *Adaptive Intelligence*—prompt response with self-adaptive learning to handle the changing circumstances happening in the city.

Among the existing physical infrastructure, water supply networks are of the utmost importance because water is the city’s most valuable resource. Efficient management of water supply networks is a key challenge faced by smart cities [3]. According to European Environment Agency [4], water leakage accounts for more than 20% of the water supply in urban water networks in most countries. With the proposed concept of smart cities [3], centralized management is expected to be enabled with real-time information collected from sensors deployed in strategic locations along the water supply networks [2,5]. In case there is any water leakage [6], we shall get an immediate alert from the monitoring platform [7,8]. In addition, real-time information from automatic meter reading (AMR) helps us to improve water conservation [9].

An important device in the management of water supply networks is the water meter, which is used to measure the volume of water supplied from a public water distribution system to a residential or commercial building [10]. In general, there are three types of water meters, namely mechanical water meters, electromechanical water meters and fully electronic water meters. Table 1 briefly summarizes advantages and drawbacks of these three major types of water meters.

Most countries adopt fully mechanical water meters due to their low cost and good reliability. However, they require labor intensive manual meter reading. For fully mechanical meters, the measurement of water flow can be based on velocity or displacement. For the former, it features impeller-based meters and turbine-based meters; for the latter, it usually includes oscillating piston and nutating disc meters.

In the past two decades, electronic circuit components were progressively integrated into mechanical water meters to provide automatic functionalities, such as AMR. These are known as the electromechanical water meters [11,12], whose measurement basis is still mechanical.

Recently, fully electronic water meters were designed using new measurement principles, such as electromagnetic [13], fluidic [14] and ultrasonic meters [15]. The electromagnetic method is based on the principle that the induced electromotive force produced by the fluid through a magnetic field is proportional to the fluid velocity. The fluidic method makes use of the $Coand\overset{˚}{a}$ *effect*—an oscillation frequency established in a fluid path with specific structure is proportional to the fluid velocity [14]. Ultrasonic water meter uses one or more ultrasonic transducers to send ultrasonic sound waves through the fluid to detect its velocity. In general, fully electronic water meters provide higher measurement accuracy as compared to fully mechanical ones, resulting in a promising meter candidate to improve water supply management in smart cities.

This paper focuses on the design and implementation of a smart water meter using a water turbine generator to realize both flow measurement and power generation. The contribution of this paper is threefold: (i) Firstly, it proposes to use a water turbine generator as both a flow measurement sensor and a power generator. In this way, it eliminates the costly external power supply or tedious battery replacement. Wherever there is water supply, it can enable real-time information collection on the water usage. (ii) Secondly, the proposed design is scalable. One can vary the dimensions of the water turbine generator to fit the corresponding water pipes. The calibration can be done online by remotely updating the digital signal processing algorithm. (iii) Lastly, the proposed smart meter can be applied to other fluids, such as air, petrol or milk. As long as the viscosity of the fluid is not high enough to degrade the performance of the turbine generator, the proposed smart meter will work.

The rest of the paper is organized as follows. Section 2 presents the related work. Section 3 presents the design and implementation of the proposed smart meter. Section 4 discusses the experimental results, followed by our conclusions and future work in Section 5.

## 2. Related Work

Modern water meters evolved from fully mechanical ones based on velocity or displacement measurements. For example, single jet meters, multiple jet meters and Woltman meters are based on velocity measurements; oscillating piston meters and nutating disc meters are based on displace measurements.

### 2.1. Velocity Measurement Meters

**Figure 1:**
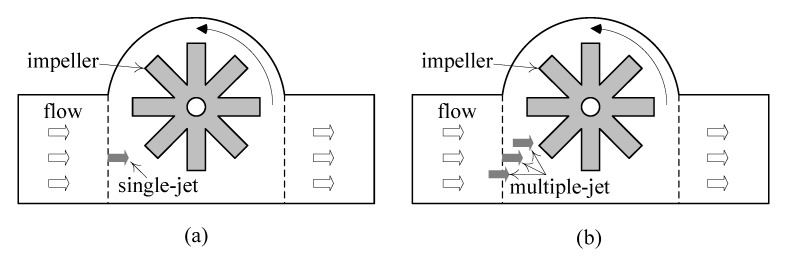
(**a**) single jet meters; (**b**) multiple jet meters.

As shown in Figure 1, jet meters are based on the tangential incidence of a single jet (or multiple jets) over a radial-vaned impeller placed inside the meters [16]. Consequently, the angular velocity of the impeller is proportional to the circulating water flow rate.

Similar to jet meters, Woltman meters use a turbine to measure the water velocity and mechanically calculate the flow rate, thus both measurement accuracy and long-term stability are guaranteed [17].

### 2.2. Positive Displacement Meters

Positive displacement meters, including oscillating piston meters and nutating disk meters, measure the water volume by dividing it into fixed volumes [18]. Oscillating piston meters measure the water volume by counting the number of times a chamber of a known volume is filled and emptied, with the aid of a rotating piston in an eccentric motion around the chamber axis of a meter. Nutating disc meters are similar to oscillating piston meters, except that the oscillating piston is replaced by a nutating disc.

### 2.3. Electronic Meters

**Figure 2:**
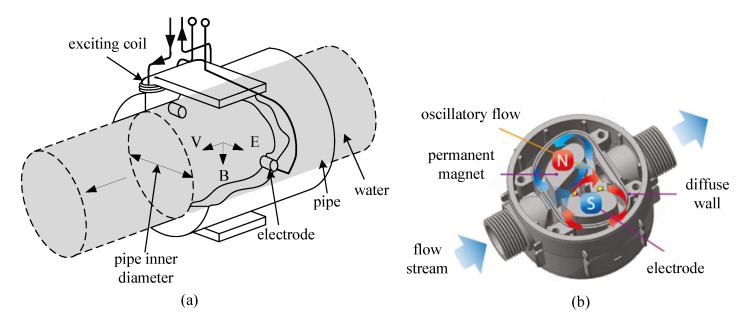
(**a**) electromagnetic meters; (**b**) fluidic effect meters.

Electronic meters include electromagnetic meters, fluidic effect meters and ultrasonic meters. As shown in Figure 2a, electromagnetic meters are based on Faraday’s law [19], and they work only with conductive fluids. For proper measurement of water flow using electromagnetic meters, the required conductivity should be greater than approximately 5 μS/cm. The volumetric flow rate of circular pipe is estimated by

(1) $$Q=\pi {\left(\frac{D}{2}\right)}^{2}v=\frac{\pi D^{2}}{4kl}\left(\frac{E}{B}\right),$$

where *D* is the inner diameter of the pipe, *v* is the flow velocity, *k* is a constant, *l* is the length of the conductor, which is usually approximated by the distance between the two electrodes as *D*, *E* is the induced voltage, and *B* is the intensity of the magnetic field that surrounds the flow.

As shown in Figure 2b, fluidic effect meters require a specially designed chamber to create a fluctuating pressure sequence that causes the water flow to oscillate [14]. The fluidic oscillator is classified into two different groups—*wall attachment devices* and *jet interaction devices*. The former is based on a phenomenon known as the $Coand\overset{˚}{a}$ *effect*, formed by the attachment of a fluid jet to an adjacent wall; and the latter usually consists of a nozzle, a bi-stable diffuser and two feedback channels. During this process, electrodes are placed to detect magnetic force and estimate the water flow rate.

Ultrasonic meters [15], including transmit time meters and Doppler effect meters, adopt ultrasonic sensors to measure water flow.

### 2.4. Smart Water Meters

Most fully mechanical water meters adopt magnetic coupling to hermetically separate the reading counter from the water flow chamber. These are called dry dial meters [20]. On the contrary, wet dial meters have the reading mechanism completely immersed in the water, which eliminates the magnetic coupling. Dry dial meters are popular, but they are more vulnerable to interference or locking by a strong magnetic field.

Thanks to the magnetic coupling mechanism, one can actually use a *Hall effect* sensor to detect the rotation of those magnets inside a dry dial meter. For example, dual complementary *Hall effect* sensors were used to develop a smart water meter while reducing the effect of environmental disturbance signals in [21]. Moreover, a magnetometer can achieve the same function [22]. Major water meter manufacturers also supply tailored water meters [23], which purposely reserve a hole for a magnetic probe and allow for quick conversion from a fully mechanical water meter to an electromechanical one.

Obviously, smart meters require electricity to power the electronic circuits. In the literature, most designs adopt fixed wired power supply or replaceable batteries. A self-powered measurement sensor was proposed in [24]; however, it is limited to small pipes with indoor use. In a separate study, a direct current (DC) motor was adopted for power generation [10]. However, it has a critical problem because it requires a hole in the pipe to connect the blades and the DC motor. In [25], Cho et al. presented a smart meter powered by electromagnetic and piezoelectric energy harvesters. However, the design requires modification of a large water pipe and it is not possible for a small water pipe. In addition, its performance will be adversely affected if the water quality is low. In [26], a platform was proposed to monitor various parameters of water quality with energy harvesting, which include pressure, temperature, pH, conductivity, flow rate and micrometric deposit thickness. The proposed platform could provide excellent sensing resolution at the expense of costly hardware components and large current consumption.

## 3. Design of a Self-Powered Smart Water Meter

Enlightened by the idea of single jet meters and energy harvesting from electrical motors [10], we design and implement a self-powered smart water meter. The key component is a micro-hydro water turbine generator (WTG), which functions as both the flow sensor and the power generator. A microcontroller is used to analyze the generated voltage to deduce the water velocity, and then the flow rate. To enable AMR, a Bluetooth module is integrated to send the real-time water flow information to a smart phone.

### 3.1. Block Diagram

**Figure 3:**
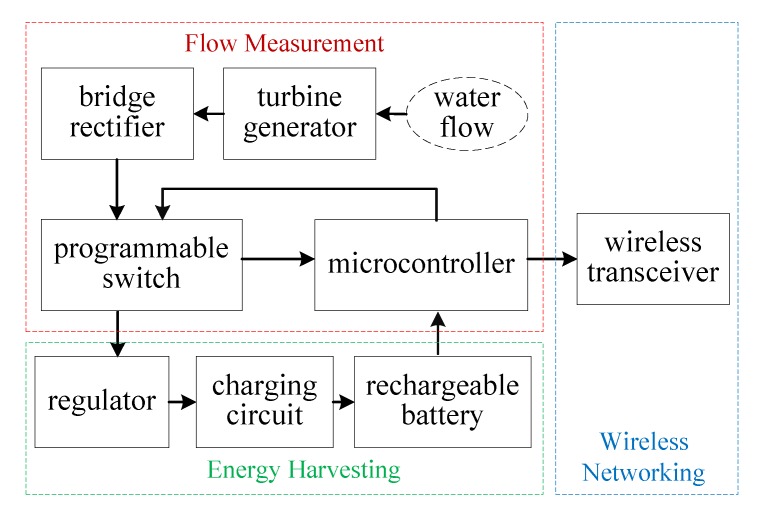
Block diagram of the smart meter.

Figure 3 shows the block diagram of the smart meter. The WTG produces the voltage signal when water goes through the WTG. The voltage signal is passed through a rectifier to a programmable single-pole double-throw (SPDT) switch. When the microcontroller performs sampling and flow rate estimation, the voltage signal is connected to microcontroller; otherwise, the voltage signal is passed to the voltage regulator, followed by the charging circuit and rechargeable batteries. The microcontroller is powered by the rechargeable batteries. When they are fully charged, they should be disconnected from the charging circuit. The smart meter can transmit real-time flow rate information through a wireless transceiver to a sink node. Note that a power management module can be included in the smart meter so that sleep mode can be introduced for energy conservation.

The design starts from selection of key input/output (I/O) components. We choose to adopt Bluetooth as the wireless networking technology due to its low power consumption. It generally requires a voltage supply at 3.3 V. The current consumption is about 40 mA, 8 mA and 2 mA for pairing, normal and sleeping mode operation, respectively. Thus, the peak power requirement of the Bluetooth module is 132 mW. With all the necessary I/O components, we can choose the microcontroller, which requires a voltage supply of 3–5 V. The power consumption is about 5 mW. Thus, the total power consumption is around 137 mW. We notice that household water usage is not evenly distributed across the day, and sometimes the flowrate is lower than 5 liters per hour (L/h) [27]. Thus, we decide to include rechargeable battery to drive the smart meter, and have chosen a 12 V 10 W WTG for our design.

### 3.2. Water Turbine Generator

**Figure 4:**
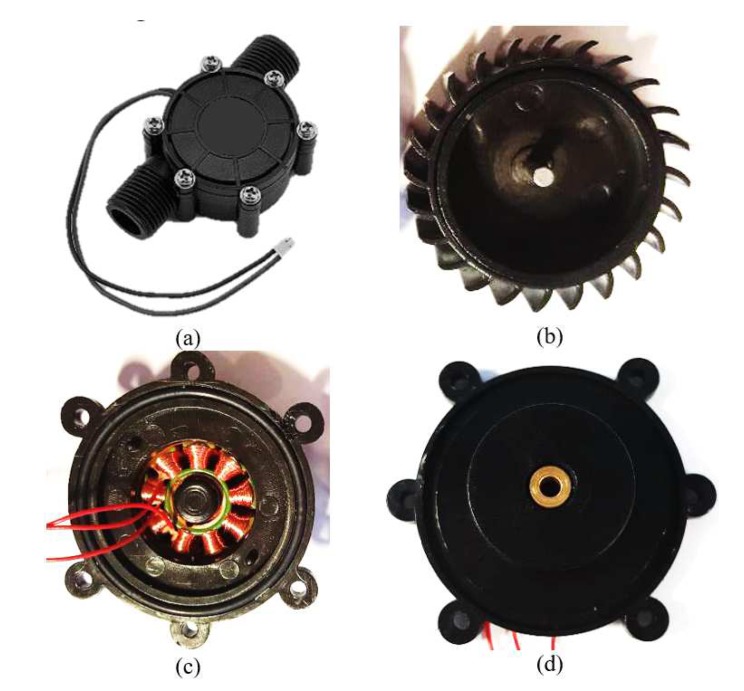
(**a**) outlook of the water turbine generator; (**b**) rotor; (**c**) stator and its corresponding wire windings as nine coils; (**d**) rotor bearing.

Figure 4a shows the outlook of the WTG, which has two major parts, the rotor and the stator, as shown in Figure 4b,c respectively. The rotor is hermetically separated from the stator. The center of the rotor is a magnetic rod, around which the magnetic field is in equilibrium, enabling the rod to float in its bearing shown in Figure 4d. This allows for frictionless motion of the rotor and essentially increases energy efficiency of the turbine.

As we know, a typical three phase voltage generator has three coils of wires. In the WTG, the stator has nine coils in three sets of coils. Each set of coils are connected in series with one end as the phase voltage output. The other ends of the three phases are connected together as a neutral ground. This configuration allows for a longer length of wire per phase, and thus a higher generated voltage. For example, one can increase the generated voltage by increasing the length of wire per phase; however, the diameter of the wire should be decreased as the total space is limited, leading to a reduced maximum current rating.

**Figure 5:**
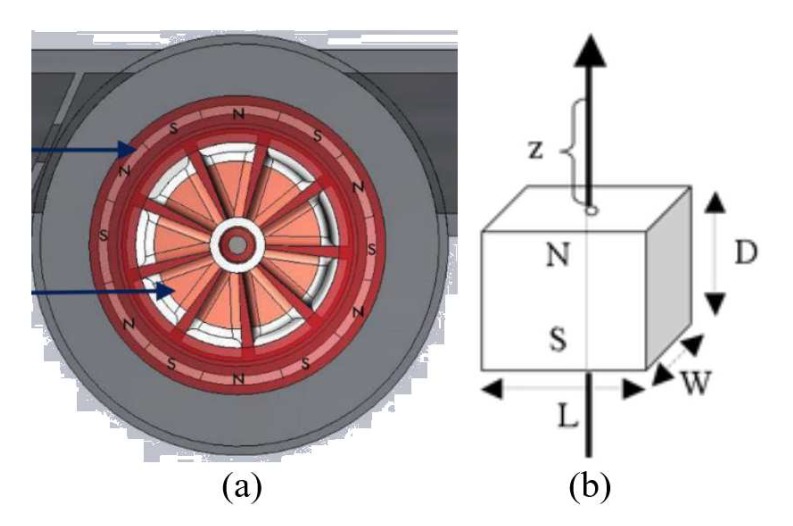
(**a**) magnet layout of water turbine generator; (**b**) block magnet model.

With a Gauss meter, we measured the strength of the magnets used in the WTG and the strongest B field is 115.8 mT. Subsequently, we used iron filings to find out the layout of the magnets, which is shown in Figure 5a. The magnet in the turbine generator is split into segments of opposite poles. Inside the stator of the generator, there is ferrite inside each coil, which causes the flux to flow through the coils. As the magnet spins with the turbine, the flux lines are disturbed and the polarity of the flux lines within the coils is reversed. This change of flux induces sinusoidal current. To increase the flux density of the turbine, a stronger magnet can be used. The flux density of a magnet can be modeled by its shape. In general, there are four types of magnet shapes, namely block, cylindrical, ring, and spherical. The magnet in the WTG is ring-shaped. The magnet is constructed of multiple magnets that most closely resemble block magnets as shown in Figure 5b. Theoretically, the magnetic field of a block magnet is modeled by

(2) $$B=\frac{B_{r}}{\pi }[\begin{array}{c} \tan^{-1}(\frac{LW}{2z\sqrt{L^{2}+W^{2}+4z^{2}}})\\ -\tan^{-1}(\frac{LW}{2(D+z)\sqrt{L^{2}+W^{2}+4{\left(D+z\right)}^{2}}}) \end{array}],$$

where $B_{r}$ is the saturation remanence, *z* is the distance from a pole face on the symmetry axis; L,W and *D* are the length, the width and the thickness of the block magnet, respectively. The dimensions of each segment of the magnet in the WTG are measured as *L* = 6.83 mm, *W* = 11 mm and *D* = 1 mm. With *z* = 0.1 mm and *B* = 115.8 mT, the $B_{r}$ is calculated to be 1.09 T, which indicates the magnet as *Sintered Neodymium Iron Boron*, *Samarium Cobalt* or *Aluminium Nickel Cobalt*. Notice that this estimation involves instrumental errors. Nevertheless, it does provide a guideline on how to choose a magnet to increase voltage generation compared to the existing magnet.

### 3.3. Voltage Generation

By Faraday’s Law of Induction, the voltage induced is proportional to the number of identical turns of a tightly wound coil of wire and the rate of change of magnetic flux through a single loop. The turbine velocity greatly affects the voltage generated by the generator. The higher the rotation speed, the higher the voltage generated. The amount of available power can be modeled by

(3) P=ηρghQ,

where η is the turbine efficiency (0.5 for typical small turbine), ρ is the water density (10^3^ kg/m^3^), *g* is gravitational acceleration (9.8 m/s^2^), *h* is the sum of the pressure head and the velocity head and *Q* is the flow rate in m^3^/s. The water pressure of households is ideally 50 psi, which is equivalent to a pressure head of 35 m. The turbine case consists of a plastic exterior with piping 15 mm in diameter. At the input of the rotor chamber, a nozzle is formed to concentrate the water flow and increase the water pressure. Consequently, the water passes through with a greater velocity.

The effect of water velocity is modeled by

(4) $$p=\rho Av^{2},$$

where *p*, ρ, *A* and *v* are the momentum, density, cross-section area and velocity of water, respectively. Due to momentum conversation, the higher the water velocity, the faster the turbine rotates.

**Figure 6:**
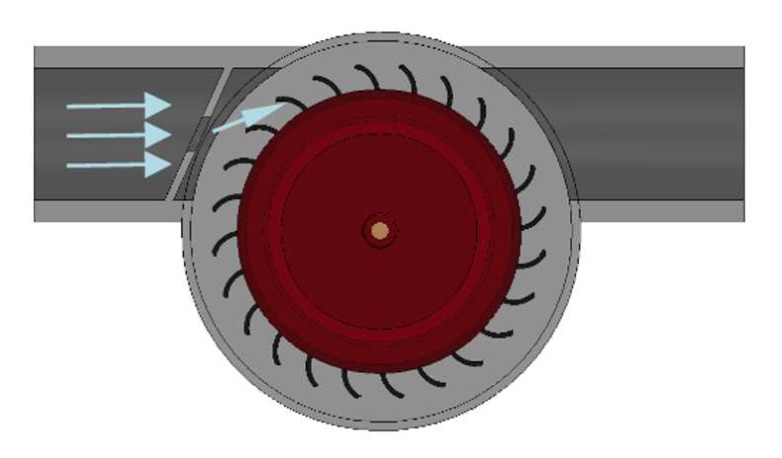
Water flow.

The chosen WTG features a single-jet impeller turbine. As shown in Figure 6, its choke point is designed at a fixed angle to change water flow direction. This allows water to be easily caught by the blades of the turbine, which are shaped in such a way to maximize the momentum transfer with maximum contact time.

The amount of available power from the WTG is dependent on the water flow rate. From the survey conducted by Watercare Service Limited (Auckland, New Zealand) [7] and water use behavior studies [27], the water usage profile in Auckland, New Zealand is summarized in Table 2. Based on the water feature compliance specifications in [24], the average *on time* of those features can be estimated as in Table 3. With the amount of water used and *on time* of each water feature, the average water flow rate can be calculated. The flow rate can then be used to calculate the possible power generated by the flow of water with Equation (3). By taking into account the *on time* of each feature, we can find the total available energy to WTG as shown in Table 4.

### 3.4. Rechargable Battery

**Figure 7:**
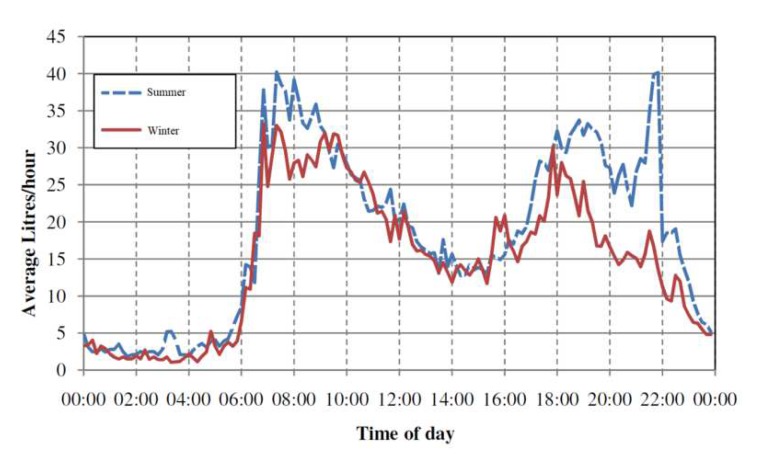
Average daily household water usage profile [27] (Copyright 2010, BRANZ).

From Table 4, we can say that the power available from water usage is enough to drive the designed smart water meter. However, the water usage profile in Figure 7 suggests that we need an energy reservoir to drive the smart meter, especially for those low usage time bands from 12:00 am to 6:00 am. Thus, a rechargeable battery is required. Theoretically speaking, a lithium ion battery is desired due to its compact size and high energy density. Despite of the limited energy available, it is possible to keep the battery at close to 63% charged with small energy consumption. With high recharge cycles, its long lifespan can justify the higher cost. However, due to a limited project budget, Nickel Metal Hydride is selected for our design.

### 3.5. Full-Wave Three Phase Rectifier

Metal-oxide-semiconductor field-effect transistors (MOSFETs) are adopted in the full-wave three phase rectifier due to their fast switching time and low $R_{ON}$. With a maximum expected current to be less than 70 mA, and typical $R_{ON}$ of around 1 to 50 Ω, the voltage drop will be roughly between 70 mV and 350 mV, which is less than half of the voltage drop of diodes.

### 3.6. Signal Processing

The voltage generated is proportional to the rotational speed of the turbine. The voltage can be probed before or after the rectifier as the measurement. If it is measured after the rectifier, voltage drop is encountered due to rectification and the accuracy might be affected. If it is measured before the rectifier, the signal has no DC component, which poses a challenging issue in the voltage measurement.

The frequency of the generated voltage of the WTG and its rotational speed in rpm is proportional. Given the number of poles as $N_{p}$, the frequency of WTG output is given by

(5) $$f=\frac{rpm\times N_{p}}{60}.$$

Due to noise and interference, it would require complex filtering techniques to detect the fundamental frequency of the generated voltage signal. Thus, it may be challenging to use frequency as measurement to deduce water flow (see Section 4 for more details).

We choose to measure the rectified voltage using the DC component. Due to the oversampling (close to MHz) of the voltage signal (with a frequency around 100 Hz), the voltage waveform can be accurately captured. The rectified voltage is supplied to both the analogue-to-digital converter (ADC) and the battery. For the sake of simplicity and energy conservation, analogue filters are not used to filter out the alternating current (AC) components of the voltage.

Digital filtering is preferred to obtain a reliable reading for the input to ADC. The main purpose of the filtering is to reduce the rectified ripple and provide a constant value of voltage, despite the fluctuation in turbine rotational speed.

The rechargeable battery may have a different internal impedance for a different degree of being charged. The internal resistance changes the load and draws different currents. From Lenz’s Law, the different currents drawn will create magnetic force of different size, requiring different force to push the turbine and change the rotation speed of the turbine.

Any influence on the turbine will degrade the accuracy of the reading and change the relationship between rotational speed of turbine with water flow. One possible solution is to model each of the responses for different loads and calibrate it with a sensor into the algorithm. Another one is to disconnect the battery charging from the turbine when an ADC sampling is in process. We have chosen the second solution in the design.

### 3.7. PCB Design

**Figure 8:**
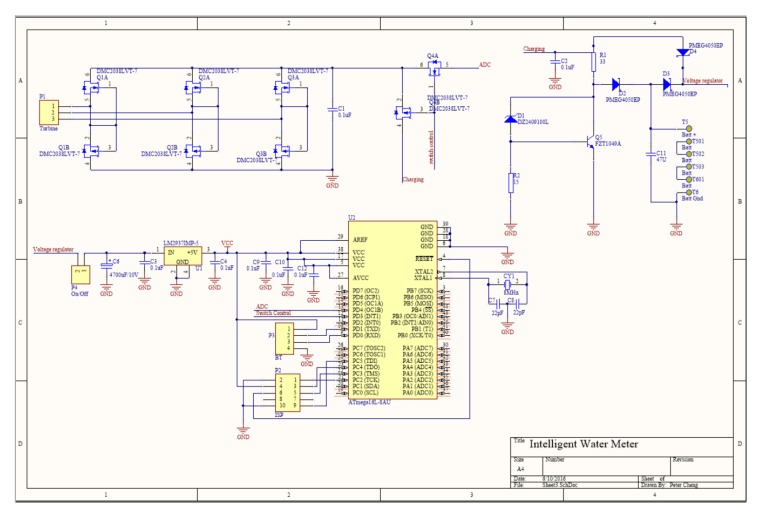
Printed circuit board schematics.

As shown in Figure 8, the main circuit uses a common microcontroller and its internal ADC to perform sampling and estimation. The input voltage is supplied through a voltage regulator. For the printed circuit board (PCB) layout, the digital section is separated from the analogue power section to maintain the quality of the signal.

A case is designed to assemble the components of the smart meter. The holder has two pieces to clamp the WTG. The case has a slot holder for the PCB with spinal reinforcement. It contains a mount location for the physical switch and a holder for the Bluetooth module. This package is designed to showcase the PCB and all parts involved. There is distance clearance between the clamp and the holder for the PCB to prevent water from making contact with the PCB. Additional circular plating is attached to shield the PCB from water splash.

### 3.8. Wireless Networking

The smart meter is equipped with a Bluetooth transceiver (HC-06 Bluetooth module). This module has a low energy consumption and it is compatible with smart phones for the module to communicate with the microcontroller using universal asynchronous receiver transmitter (UART) protocol. For wireless networking, we use a mobile phone to function as a sink node, and develop an application uses the MIT App Inventor 2 [28]. In this way, AMR can be realized.

The baud rate of the Bluetooth module can be configured from 1200 bauds/s to 1,382,400 bauds/s, with a default value of 9600 bauds/s. Next, the module draws 0.5 mA in standby mode, and 8.5 mA in active mode. It requires about 35 mA in pairing mode. To reduce energy consumption, we can set the baud rate to a lower value as the required data are not high for a water meter. For example, one meter reading per hour should be adequate. In addition, it is better to fix the paired device to the meter so that it can eliminate extra energy consumption due to frequent pairing.

We noticed that Bluetooth has short communication distance, but it does offer low energy consumption. If longer communication distance is necessary, Wi-Fi is a better candidate. Existing smart meters mostly use cellular radios, but they lead to extra operational cost of monthly subscription fee paid to telecommunication operators.

## 4. Experiments and Results

This section presents the experiment set up and results.

### 4.1. Test Apparatus

A water tank and a water pump (Aquapro AP3000, Forrestdale, Australia) are used to emulate residential water supply. The maximum water flow for the pump is 3000 L/h, which is approximately the residential water flow rate. In this way, the test unit will only require a fixed amount of water, thus vastly reducing water usage [29]. In addition, we can simulate the water usage profile as plotted in Figure 7 by controlling the pump switch. A commercial water meter (Super-Rite K24 made by Jerrycar, Frankfort, South Africa) was used to calibrate the smart water meter designed.

### 4.2. Meter Calibration

Water pressure in pipes is varying constantly. These inconsistencies are unpredictable and can be caused by many factors, including air bubbles and pressure build up. We aim to find a relationship with water flow rate and the generated voltage.

**Figure 9:**
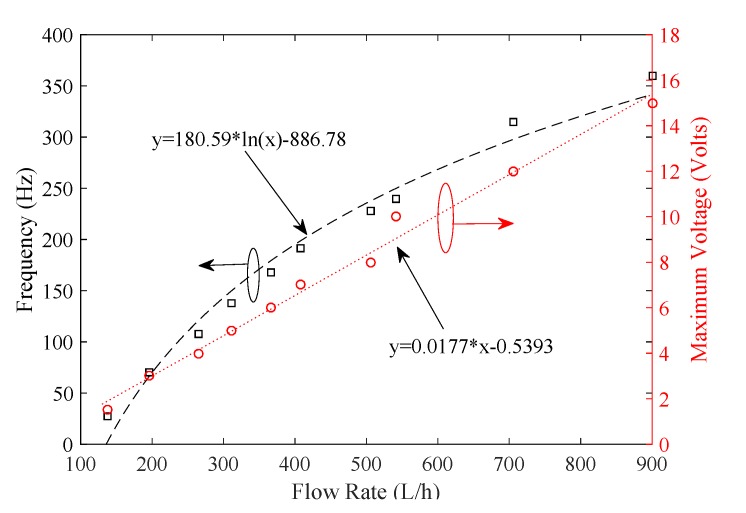
Voltage and frequency behavior of generated voltage.

As shown in Figure 9, the frequency and peak voltage are plotted against the water flow rate. The frequency of the generated voltage appears to be more sensitive to inconsistencies, making it difficult to determine the relationship accurately. Thus, it is a better choice to use voltage values to estimate the flow rate.

**Figure 10:**
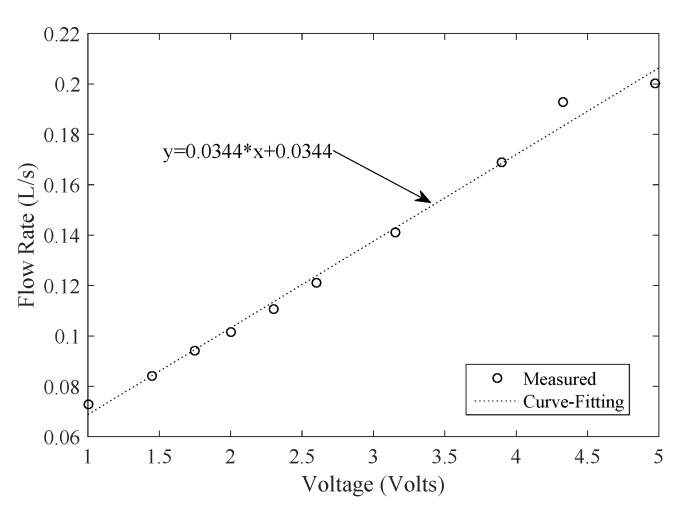
Voltage and flowrate relationships.

**Figure 11:**
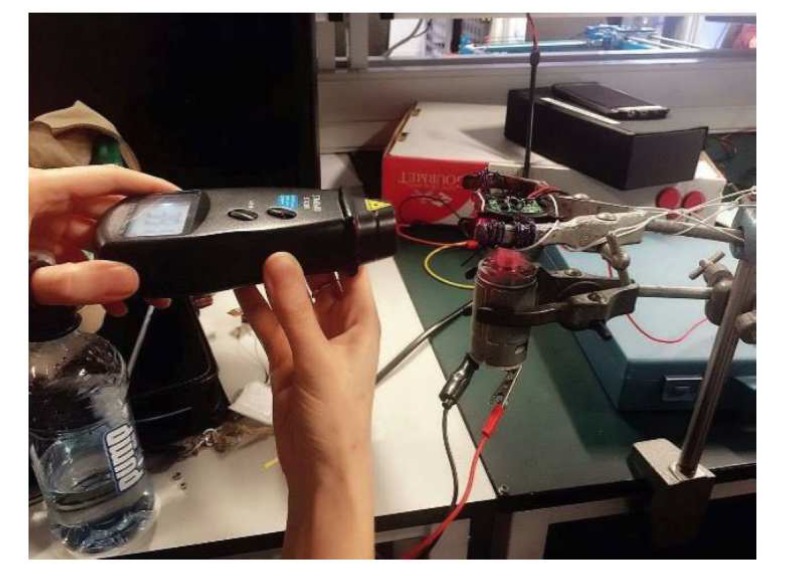
Tacho meter measuring RPM of a turbine spun by a DC motor.

**Figure 12:**
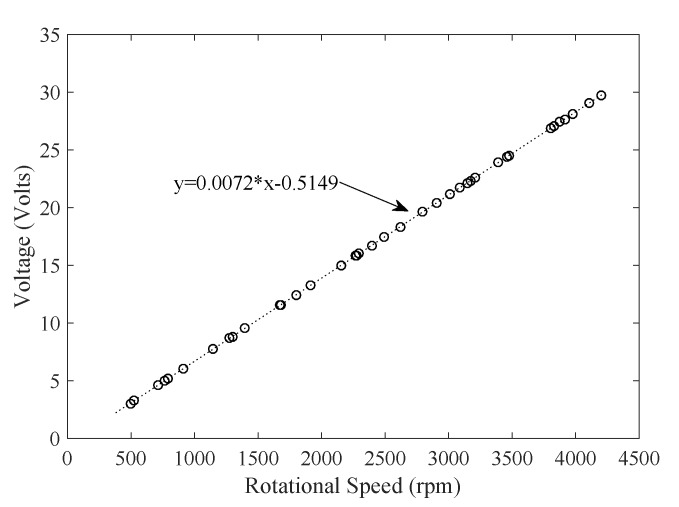
Test results for voltage and RPM.

As shown in Figure 10, the voltage and flow rate show a strong proportional relationship. The suitable flow rate measurement range is [200 650] L/h. Furthermore, as shown in Figure 11, a DC motor with a suction cup is used to spin the turbine. The rotational velocity in revolutions per minute (RPM) of the turbine is measured with a tachometer. The values of rotational speed and generated voltage are plotted in Figure 12.

As shown in Figure 12, the relationship is highly linear. The negative offset can be due to the energy lost in friction and motion. By curve fitting, an equation is found and coded in the software algorithm to determine the water flow rate of the meter.

Next, calibration is performed to compare our linear model with real measurements. The test process is to let water run through the water turbine for a fixed amount of time into a measurement jug, then compare the recorded value of the water meter with the actual amount of water. The algorithm of the smart meter is adjusted to minimize the difference. This process is repeated until the difference is smaller than a predefined threshold.

### 4.3. Smart Metering Algorithm

**Figure 13:**
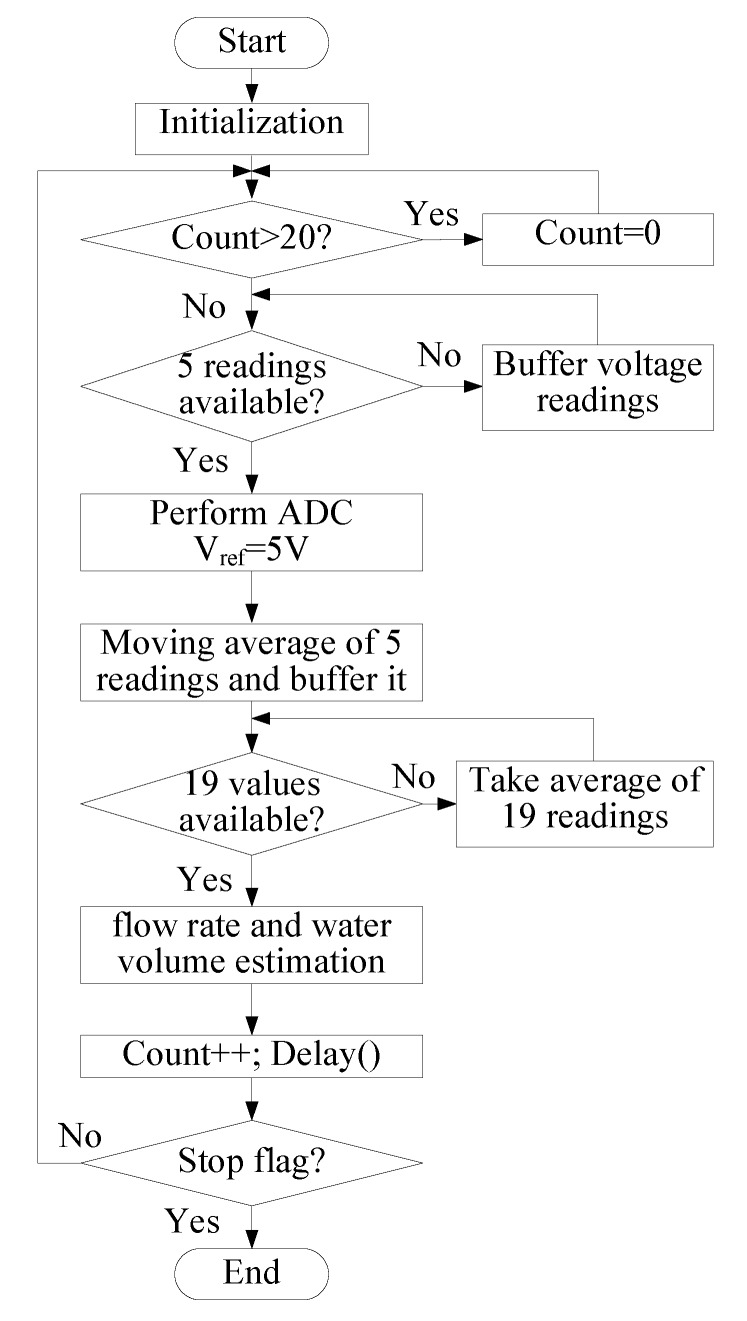
Smart meter algorithm flowchart.

Figure 13 shows the process of smart metering. It takes an analogue signal reading into the ADC, converts that value into a voltage value, then performs two sets of moving averages to filter out the high frequency components. The value is then used to estimate the flow rate. Finally, the flow rate is integrated over time to estimate the total water volume.

### 4.4. Comparison with Existing Smart Meters and Mechanical Water Meters

Table 5 compares our proposed design with existing smart meters. In addition, Table 6 compares the proposed design with a traditional mechanical water meter.

In [30], Alrowaijeha and Hajj presented a proof of concept of a self-powered water meter using a micro hydro turbine. After a careful comparison between our work and theirs, we identified the following key differences:

- We presented a complete design of the smart meter, including a customized printed circuit board using a microcontroller, a digital signal processing algorithm, and analysis of the design of a water turbine generator, while, in [30], the authors presented their design using the off-the-shelf products without alteration, such as a micro hydro turbine with DC output, charging module, battery, single-board computer (Raspberry Pi 3 Model B), Data Acquisition System (DATAQ instrument unit).
- We did not directly use the off-the-shelf water turbine generator. Instead, we removed its internal circuit board, used the existing coil windings and collected the generated AC signal to feed our customised PCB for signal processing and energy harvesting. However, the authors in [30] used the generated DC signal from an off-the-shelf micro hydro turbine. Therefore, we derived empirical formulas between the amplitude (and the frequency) of the generated voltage and water flow rate. However, no such information is provided in [30].
- We presented the analysis of water usage of a typical New Zealand house to show that the amount of energy to be harvested is adequate to power the smart meter. However, in [30], there is no such analysis.
- Our design is based on a 10 W–12 V water turbine generator with an inlet/outlet diameter of 15 mm, while, in [30], they used a 10 W–80 V water turbine generator with inlet/outlet diameter of 12.7 mm. The resulted measurement range is different. For our work, the measurement range is [200 650] L/h, and, for [30], it is [200 350] L/h.

As mentioned in [33], it was mentioned that the consumed water head cannot exceed 5 m to avoid negative effects on the normal water supply through the whole urban area. In this paper, the proposed design was intended for a household application, for which slightly higher water head loss can be tolerated. Nevertheless, to avoid excessive water head loss, we can vary the choke point of the water turbine generator to adjust the water injection angle. In addition, we can also use a multiple-jets impeller turbine instead of the single-jet impeller turbine.

## 5. Conclusions

A self-powered smart water meter is designed using a turbine generator with a Bluetooth transceiver, which features a cost effective solution to water resource management in smart cities. For the implemented prototype, the suitable flow rate measurement range is [200 650] L/h. A mobile app is developed to deliver real-time information to a user’s smart phone. In addition, calibration of the smart meter can be performed by changing the variables in the modeling equation so that it allows for other liquids and piping for industrial applications such as brewery, refineries and bakery. In addition, other sophisticated sensors can be added to the turbine to extend its abilities beyond measuring flow and quantity.

The current prototype of the smart meter has three limitations: (i) It has difficulty in detecting small water flows (e.g., less than 200 L/h) that are not powerful enough to push the turbine to spin. This is particularly true for leak detection, preventing it from being a strong competitor to the traditional mechanical meters. One possible solution is to adopt oscillating piston or nutating disk (i.e., measurement mechanism in positive displacement meters) to drive the rotor of the proposed smart meter. (ii) Large water flow poses another challenge due to the magnet saturation and the turbine slip, in which the water flow escapes around the turbine and no longer contributes to the rotational speed of the turbine. Thus, it is hard to accurately measure water flow above a certain threshold. Magnets with stronger magnetic field can be chosen to mitigate this situation. (iii) Bluetooth module has a short communication range, which limits the distance between the smart meter and its sink node. Other wireless technology with a longer communication range can selected. Our future work will investigate these options and improve the current design of the self-powered smart meter.

In addition, in this paper, we have assumed that water from the urban water supply network has a constant of density and gas volume fraction. Abrupt change in the water density will affect the accuracy of the proposed water meter. For example, increased gas volume fraction will reduce the water density, leading to lower generated voltage from a water turbine generator. To deal with this challenge, an extra photoelectric water density sensor could be added to provide feedback information to the microcontroller adopted in this design, which would adjust its algorithm to compute the water volume based on the generated voltage in a real-time manner. This is part of our future work on this topic.

## Figures and Tables

**Table 1:** Comparison of water meters.

Types	Advantages	Drawbacks
Fully Mechanical	simple design, low cost reliable operation	narrow measurement range, reduced accuracy at low flow rates cumulative measurement only, lack of real-time information
Electromechanical	real-time information	requires extra protection for the electronic component reduced stability
Fully Electronic	high accuracy, real-time information	requires extra waterproof protection and power supply

**Table 2:** Water usage profile in Auckland, New Zealand.

	Summer	Winter
Average	179 lpd	174 lpd
Median	143 lpd	130 lpd
Washing machine	21%	24%
Shower	24%	30%
Toilet	18%	19%
Tap	11%	16%
Leak	4%	2%
Outdoor	17%	6%
Bathtub	2%	1%
Dishwasher	1%	1%
Misc	0%	1%

* lpd denotes litres per day.

**Table 3:** Water daily usage time for different features (seconds).

Feature	Summer	Winter
Bath	11.93	34.8
Shower	429.6	261
Sink	98.5	92.8
Laundry tub	35.8	17.4
Wishing Machine	187.95	208.8
Dishwasher	8.95	8.7
Toilet	161.1	165.3

**Table 4:** Available power from household water usage.

Feature	Summer	Winter
Power	32.87 W	37.92 W
Energy	30,698.5 J	29,841 J

**Table 5:** Comparison of smart water meters.

Types	Advantages	Disadvantages
[23]	simple design, reliable operation	customized design, external power supply
[24]	self-powered, light-weight	indoor use only, small pipes
[25]	self-powered, hybrid energy harvesting	complex design, large pipes only
[10]	self-powered, simple design	requires pipe holes, limited reliability
[31]	self-powered, IoT connectivity	requires pipe holes, large pipes only
[32]	self-powered, realiable operation	complex design, separate energy harvesting unit
This Work	self-powered, scalable design	requires water proof, customized design

**Table 6:** Comparison of the proposed smart meter and traditional mechanical water meter.

Types	Advantages	Disadvantages
Mechanical Water Meter	simple design, reliable operation large measurement range	requires manual reading no AMR functionality, no remote monitoring
This Work	self-powered, scalable design AMR functionality real-time monitoring	requires moisture protection narrow measurement range
